# Cordycepin attenuates NLRP3/Caspase-1/GSDMD-mediated LPS-induced macrophage pyroptosis

**DOI:** 10.3389/fphar.2025.1526616

**Published:** 2025-02-14

**Authors:** Zige Liu, Li Lv, Jiao Wei, Yuli Xie, Mujia Jili, Yian Huang, Rirong Yang, Yu Luo

**Affiliations:** ^1^ Center for Genomic and Personalized Medicine, Guangxi key Laboratory for Genomic and Personalized Medicine, Guangxi Collaborative Innovation Center for Genomic and Personalized Medicine, University Engineering Research Center of Digital Medicine and Healthcare, Guangxi Medical University, Nanning, Guangxi, China; ^2^ Department of Physiology, School of Basic Medical Sciences, Nanning, Guangxi, China; ^3^ Department of Immunology, School of Basic Medical Sciences, Guangxi Medical University, Nanning, Guangxi, China; ^4^ Department of Clinical Laboratory, Guangxi Academy of Medical Sciences, The People's Hospital of Guangxi Zhuang Autonomous Region, Nanning, Guangxi, China

**Keywords:** cordycepin, macrophage, macrophage pyroptosis, LPS, RAW 264.7

## Abstract

Pyroptosis, a form of programmed cell death driven by the NLRP3 inflammasome, is a key contributor to inflammation in various diseases. This study aimed to investigate the anti-inflammatory mechanisms of cordycepin, focusing on its role in macrophage pyroptosis. Molecular docking analysis was performed to evaluate the binding affinity of cordycepin to key pyroptosis-related proteins, including NLRP3, Caspase-1, and GSDMD. RAW264.7 cells were pre-treated with cordycepin to assess its effects on pyroptosis. Key measurements included reactive oxygen species (ROS) levels, xanthine oxidase (XO) activity, and the expression of NLRP3, Caspase-1, and GSDMD. Additionally, lactate dehydrogenase (LDH) release, interleukin (IL)-1β and IL-18 levels in the culture supernatant, and macrophage cell death rates were evaluated using Hoechst 33342/PI dual staining. The results demonstrated that cordycepin exhibits strong binding affinity for NLRP3, Caspase-1, and GSDMD. Cordycepin pre-treatment significantly reduced ROS levels and XO activity, inhibited the expression of NLRP3, cleaved-Caspase-1, and cleaved-GSDMD, and decreased pyroptosis-associated inflammatory cytokines IL-1β and IL-18, along with Caspase-1 activity. Furthermore, cordycepin reduced the macrophage pyroptosis rate. In conclusion, cordycepin inhibits macrophage pyroptosis by reducing XO activity, suppressing ROS production, and regulating the expression of key molecules in the NLRP3/Caspase-1/GSDMD pathway. These findings provide a strong experimental basis for the potential development of cordycepin as a novel anti-inflammatory agent.

## Introduction

Inflammation is a complex biological response of body tissues to microbial infection, external stimuli, or damage to tissues. As it is a protective response involving immune cells and molecular mediators, inflammation plays a key role in the overall innate immune response (Soares et al., 2023). Appropriate inflammation plays a protective role by recruiting immune cells and inflammatory cells to locally eliminate pathogens. However, excessive inflammation can lead to over-expression of inflammatory cytokines, leading to inadvertent activation of immune cells in the surrounding environment. This has the potential to trigger host tissue damage and eventually autoimmune disease pathologies including inflammatory bowel disease, sepsis, and rheumatoid arthritis (Yu et al., 2022; Yuan et al., 2023). Therefore, inhibiting any prolonged over-production of inflammatory cytokines is likely key to alleviating/preventing many inflammation-based autoimmune diseases.

Because macrophages are an important immune cell in the innate immune response, it is not unexpected that these cells are widely distributed in various tissues. Macrophages not only can engulf and kill bacteria/other pathogens, but they can also perform immune regulation and antigen presentation functions. In addition, macrophages can specifically recognize pathogens through their surface pattern recognition receptors, thereby sensing the invasion of pathogenic micro-organisms or tissue damage and inducing the body to produce an immune response. However, there are events whereby macrophages can be forced into a ‘death state’ and this impacts on the overall host immune response. Specifically, pyroptosis is a highly inflammatory form of programmed cell death that can result in the macrophages secreting a wide variety of inflammatory cytokines, thereby triggering a strong host inflammatory response (Kesavardhana et al., 2020; Wang et al., 2020; Wang and Sun, 2022). Thus, gaining insight into the mechanism of macrophage pyroptosis could provide investigators a more complete understanding of the pathogeneses of many inflammation-based diseases. To this end, understanding how known anti-inflammatory agents might impact on this process in macrophages may contribute to this increased insight.

Cordycepin - the main active ingredient isolated from *Cordyceps militaris* (belonging to the purine alkaloids) - is a natural nucleoside analogue (Wang et al., 2022). Over a wide range of studies, it has been shown that cordycepin can impart various pharmacological effects and biological activities, including anti-tumor, immunoregulatory, anti-inflammatory activities (Radhi et al., 2021). In several *in vivo* studies, the impact of cordycepin on macrophages/related macrophage-driven pathologies was evaluated. In one study, cordycepin was found to improve the inflammatory state associated with intervertebral disc degeneration in rat, in part, by its ability to inhibit activation of macrophages and their infiltration into affected tissues (by regulating NF-κB signaling pathways) (Li et al., 2016). In another study, in a complete Freund’s adjuvant (CFA)-induced mouse paw inflammation model, cordycepin was shown to significantly inhibit localized secretion of inflammatory factors such as tumor necrosis factor (TNF)-α, interleukin (IL)-1β, and IL-6 in the inflamed mouse paw, thereby reducing the aggregation of macrophages in the area (Yang et al., 2020; Cheng et al., 2017) found that cordycepin could impart neuroprotective effects in a rat model of cerebral hemorrhage, and that this effect was due, in part, by an inhibition of the activation of NLRP3 inflammasomes. Despite these findings, the precise molecular mechanisms by which cordycepin modulates NLRP3 inflammasome activation and pyroptosis remain poorly understood. The NLRP3 inflammasome’s role as a central hub in inflammatory signaling suggests that cordycepin’s effects on this pathway could be pivotal in mediating its anti-inflammatory properties. Investigating these interactions may provide deeper insights into cordycepin’s therapeutic potential.

Even with those findings, specific molecular mechanisms by which cordycepin inhibits macrophage pyroptosis remain unclear. Among the pathways open to investigation are cordycepin effects potentially mediated by the NOD-like receptor family pyrin domain containing 3 (NLRP3) inflammasome. Although previous studies have explored the anti-inflammatory effects of cordycepin, detailed mechanistic studies focusing on its role in inhibiting macrophage pyroptosis are still lacking. In particular, the interaction of cordycepin with key pyroptosis-related proteins and its regulatory effects on the NLRP3 inflammasome pathway require further investigation. Accordingly, in the study reported here, mouse RAW264.7 cells were used as a model macrophage line. The goal here was to establish both an LPS (lipopolysaccharide) - induced NLRP3 inflammasome activation model and an LPS + ATP (adenosine triphosphate)- induced macrophage pyroptosis model, to investigate the potential effects of cordycepin on NLRP3 inflammasome-mediated macrophage pyroptosis. In parallel, to gain a better understanding of how cordycepin might interact with macropahges, binding of cordycepin with key proteins such as NLRP3, Caspase-1, and GSDMD was evaluated using molecular docking technology.

## Materials and methods

### Molecular docking of cordycepin with pyroptosis-critical target proteins

The 2-D structure of cordycepin was downloaded from the PubChem database and imported into ChemBio3D software (in order to save the cordycepin 3-D structure in mol2 format). Thereafter, a search for potential key target proteins, such as NLRP3, Caspase-1, and GSDMD involved in cell signaling pathways, was undertaken in a PDB database. Once confirmed as potential targets, 3-D structures of these target proteins were also downloaded. PyMOL 1.8. × software was then used to remove water molecules, ligands, add hydrogen atoms, and to modify charges to the 3-D structures of the key target proteins in preparation for molecular docking analysis (Seeliger and de Groot, 2010).

Once all the structures were imported, the 3-D structure of cordycepin and the target proteins were grouped into AutoDock 4.2 software (which then converted them to pdbqt format), and molecular docking analyses was performed. In all scenarios, the binding energy of each interaction between the small molecule ligand and the protein macromolecule was recorded. All results were then visualizes using PyMOL 1.8 software.

### RAW264.7 cells

RAW264.7 cells were obtained from the Cell Bank of the Chinese Academy of Sciences (Shanghai, China) and cultured at 37°C (under 5% CO_2_) in Dulbecco’s Modified Eagle’s Medium (DMEM, Sigma) containing 10% fetal bovine serum (DMEM, Sigma), penicillin (100 U/mL), and streptomycin (100 mg/mL). Cultures were passaged every 3 days. Cordycepin (MCE, United States) is a water-soluble molecule, which facilitates its preparation and use in cell culture experiments.

### Determination of reactive oxygen species (ROS) formation

RAW264.7 cells were seeded into 12-well plates at 5 × 10^4^ cells/well (1 mL/well). After 12 h at 37°C, the wells were randomly designated as one of six groups. The groups were to be cells treated with: DMEM medium only (sham), 1 μg LPS/mL (L4391; Sigma, St. Louis, MO), 10 nM NAC (*N*-acetylcysteine; Sigma) + 1 μg LPS/mL, and low-, medium-, and high-dose cordycepin (cultures treated with LPS 1 μg/mL in combination with 25, 50, or 100 μg cordycepin/mL, respectively). Subsequently, all groups were cultured for 24 h. After the time had elapsed, the medium was removed from each well, and the cells were gently rinsed with phosphate-buffered saline (PBS, pH 7.4). Subsequently, 2′, 7′-dichlorodihydro- fluorescein diacetate (DCFH-DA; diluted in serum-free DMEM to 10 μM; D6883, Sigma) was added to each well (total volume of 1 mL/well). The cells were then incubated in the dark in a 37°C CO_2_ incubator for 30 min. The cells were then washed three times with serum-free cell DMEM to thoroughly remove any DCFH-DA that had not entered the cells. For reactive oxygen species (ROS) detection, the Varioskan ALF microplate reader (Thermo Fisher Scientific, Waltham, MA) set at an excitation wavelength of 488 nm/emission wavelength of 525 nm was used to measure the fluorescence intensity of ROS reacting with the DCFH-DA product (DCF). Additionally, the fluorescence intensity was monitored using a confocal microscope (at 100X magnification) (Carl Zeiss, Oberkochen, Germany). Representative samples were photographed to provide a qualitative record of the formation of ROS withing the cells.

### Determination of xanthine oxidase (XO) levels

RAW264.7 cells were seeded into 6-well plates at 1 × 10^5^ cells/well (3 mL/well). After 12 h at 37°C, the wells were randomly designated as one of five groups. The groups were to be cells treated with: DMEM medium only (sham), 1 μg LPS/mL, and low-, medium-, and high-dose cordycepin (cultures treated with LPS 1 μg/mL in combination with 6.25, 12.5, and 25 μg cordycepin/mL, respectively). Subsequently, all groups were cultured for 24 h.

After the exposure period, the cells were collected by centrifugation (4°C, 12,000 g, 5 min) and the resultant supernatant then carefully transferred to new 1.5-mL Eppendorf tube. For the determination of xanthine oxidase (XO) levels, an assay kit was employed (Abbkine Scientific, Wuhan, China) according to manufacturer instructions. In all cases, there were three replicate wells for each treatment sample. Before and after the kit-indicated incubation period, the absorbance at 450 nm in each well was measured using a Varioskan ALF microplate reader (Thermo Fisher Scientific). Changes in OD over the 30 min (i.e., OD_30 min_–OD_0 min_) were recorded for each group.

### Gene expression of *XO, Nlrp3, Caspsae-1, Gsdmd, IL-1β* and *IL-18*

RAW264.7 cells were seeded into 6-well plates at 1 × 10^5^ cells/well (3 mL/well). After 12 h at 37°C, the wells were randomly designated as one of six groups. The groups were to be cells treated with: DMEM medium only (sham), 1 μg LPS/mL, 1 nM Febuxostat (Sigma) + 1 μg LPS/mL, and low-, medium-, and high-dose cordycepin (cultures treated with LPS 1 μg/mL in combination with 6.25, 12.5, and 25 μg cordycepin/mL, respectively). Subsequently, all groups were cultured for 24 h. Cells in each group were then harvested by centrifugation and lysed to extract total RNA using TRIzol reagent (Invitrogen, San Diego, CA, United States) using protocols recommended by the manufacturer**.**

After confirming the concentration/purity of the isolated RNA using a Nanodrop 2000 spectrophotometer (Thermo Fisher Scientific), and the RNA purity was judged by an OD value of 260/280 (OD value was about 2.0), 1 μg total RNA/sample set was used to synthesize complementary cDNA with a RevertAid First Strand cDNA synthesis kit (Thermo Fisher Scientific) Reverse transcription-polymerase chain reaction (RT-PCR) was then carried out in a LightCycler PCR system (Roche, Basel, Switzer-land) using a SYBR Premix Ex Taq kit (TaKaRa, Tokyo, Japan) in a 10-μL RT-PCR reaction volume. The process involved initial denaturation at 95°C for 5 min, followed by 40 cycles of denaturation at 95°C for 10 s, annealing at 60°C for 15 s, and extension at 72°C for 10 s. All experiments were conducted in triplicate; *β-actin* was used as reference gene to normalize target gene expression levels. The primer sequences used are presented in Table 1. Relative expression of the target cellular mRNA was calculated using the 2^−ΔΔCT^ method.

**TABLE 1:** Primers used in RT-PCR assays.

Gene	Sequence
*Xo*	5′-TAT​GGG​GTG​GCT​TGC​TCA​GA-3′ (F)
	5′-CAA​GAC​CCT​GGA​CAA​ATG​CC-3′ (R)
*Nlrp3*	5′-GCC​GTC​TAC​GTC​TTC​TTC​CTT​TCC-3′ (F)
	5′-CAT​CCG​CAG​CCA​GTG​AAC​AGA​G-3′ (R)
*Caspsse-1*	5′-ATA​CAA​CCA​CTC​GTA​CAC​GTC​TTG​C-3′ (F)
	5′-TCC​TCC​AGC​AGC​AAC​TTC​ATT​TCT​C-3′ (R)
*Gsdmd*	5′-CGA​TGG​GAA​CAT​TCA​GGG​CAG​AG-3′ (F)
	5′-ACA​CAT​TCA​TGG​AGG​CAC​TGG​AAC-3′ (R)
*β-actin*	5′-GTG​CTA​TGT​TGC​TCT​AGA​CTT​CG-3′ (F)
	5′-ATG​CCA​CAG​GAT​TCC​ATA​CC-3′ (R)
*IL-1β*	5′-GCA​ACT​GTT​CCT​GAA​CTC​AAC​T-3′ (F)
	5′-ATC​TTT​TGG​GGT​CCG​TCA​ACT-3′ (R)
*IL-18*	5′-CCT​GAC​ATC​TTC​TGC​AAA​GG-3′ (F)
	5′-GCT​GTC​TTT​TGT​CAA​CGA​ACA-3′ (R)

### Western blot assay

RAW264.7 cells were seeded into 6-well plates at 1 × 10^5^ cells/well (3 mL/well). After 12 h at 37°C, the wells were randomly designated as one of six groups. The groups were to be cells treated with: DMEM medium only (sham), 1 μg LPS/mL, 1 nM Febuxostat (Sigma) + 1 μg LPS/mL, and low-, medium-, and high-dose cordycepin (cultures treated with LPS 1 μg/mL in combination with 6.25, 12.5, and 25 μg cordycepin/mL, respectively). Subsequently, all groups were cultured for 24 h. 200 μL of lysis buffer supplemented with protease inhibitors was added to the 1.5 mL EP tube of cells and then placed on ice for 10 min. All samples were centrifuged at 4°C for 10 min at 12,000 rpm, and the supernatant was collected in a new 1.5 EP tube for protein concentration determination. The protein concentration was quantified using BCA Protein Quantification Kit (Beyotime, Jiangsu, China). From each sample isolate, aliquots of the total proteins (containing 30 μg protein) were removed to undergo separation via 10% sodium dodecyl sulfate (SDS)-polyacrylamide gel electrophoresis. Following resolving of the proteins, all materials were transferred to a poly-vinyldene fluoride (PVDF) membrane. For each protein of interest, a separate membrane was prepared in parallel (this avoids issue of antigen loss from membrane stripping steps before a second protein might be assessed). Subsequently, each membrane was blocked by incubation in 5% skim milk in Tris-buffered saline-Tween buffer (TBS-T: 10 mM Tris-HCl, 50 mM NaCl, 0.25% Tween-20) for 1 h. The membrane was then coated with a solution of TBS-T containing primary antibody against XO, GAPDH, cleaved N-terminal GSDMD, cleaved-Caspase-1 or NLRP3, and incubated overnight at 4°C with gentle rocking. The primary antibodies used were rabbit anti-mouse GAPDH, cleaved-Caspase-1, or cleaved N-terminal GSDMD (each at 1:1,000 dilution) or anti-XO (1:500 dilution), or anti-NLRP3 (1:1,500 dilution). Each dilution was based on recommendations from the supplier (Cell Signaling Technology, MA, United States). After this incubation, each membrane underwent three washes with TBS-T. The membranes were then coated with TBS-T containing horseradish peroxidase (HRP)-conjugated anti-rabbit secondary antibody (1:5,000 dilution; Proteintech, Wuhan, China) for 1 h at room temperature. Each membrane was then again gently rinsed with TBS-T before each was placed in a solution of TBS-T for 30 min at room temperature. All signals that developed were then detected using a Bio-Rad imaging system (Bio-Rad, Hercules, CA). Gray levels corresponding to levels of each specified protein were quantified and then normalized in relation to GAPDH using ImageJ software (NIH, Bethesda, MD).

### Cell death assay

RAW264.7 cells were seeded into 12-well plates at a density of 5 × 10^4^ cells/well (1 mL/well). After 12 h of incubation at 37°C, the wells were randomly assigned to one of five groups. The groups included: cells treated with DMEM medium only (sham), 1 μg/mL LPS + 5 mM ATP (Solarbio, Beijing, China), or 1 μg/mL LPS + ATP in conjunction with low-, medium-, or high-dose cordycepin (6.25, 12.5, and 25 μg/mL cordycepin). All groups were then cultured for 24 h. Morphological changes associated with pyroptosis and pyroptotic cell death were assessed using Hoechst 33342/propidium iodide (PI) dual staining and examined under a TH4-200 fluorescence microscope (Olympus Corporation, Tokyo, Japan). PI staining indicates loss of membrane integrity and cell death. Additionally, LDH activity (which reflects cell death and the release of intracellular enzymes) in the medium collected from each well prior to staining was measured using an LDH kit (Nanjing Jiancheng Bioengineering Institute, Nanjing, China).

For the Hoechst 33342/PI double staining, the treated RAW 264.7 cells had their culture medium collected and then were rinsed with PBS before staining with 10 µL Hoechst 33,342 staining solution (part of a Hoechst 33342/PI dual staining kit; Beyotime Institute of Biotechnology, Shanghai, China) in the dark for 10 min at 37°C. The cells were then stained with 5 µL of 5 µg PI/mL solution (Beyotime) in the dark for 10 min at 25°C. After three PBS washes, images in the wells were captured using the fluorescent microscope (at ×100 magnification). ImageJ software (NIH, Bethesda, MD) was used to quantify PI-stained cells in five randomly selected microscopic fields. Levels of PI^+^ cells were calculated as: PI-stained cells (%) = (number of red fluorescent cells/total blue fluorescent cells) × 100.

To assess LDH activity in the culture medium, well supernatants (200 µL/sample) were collected and centrifuged (400 × g, 5 min, 4°C). Subsequently, 20 µL of cleared supernatant was placed in a dedicated well in a 96-well test; to each well, 5 µL kit-provided Co-enzyme I (or deionized water) and 25 µL kit substrate buffer was then added and the plate was incubated for 15 min at 37°C. Following this, 25 µL of kit-provided 2,4-dinitrophenylhydrazine solution was added to each well, and the samples were incubated an additional 15 min at 37°C. To stop the reaction, 250 µL of 0.4 M NaOH solution was added to each well, and the plate was incubated for 5 min at 25°C. The absorbance in each well at 450 nm was measured using a Varioskan ALF microplate reader (Thermo Fisher Scientific) and percentage LDH release was then calculated according to manufacturer instructions.

### Measurement of caspase-1 activity

After their respective treatments, RAW 264.7 cells were evaluated for caspase-1 activity via a caspase-1 activity assay kit (Beyotime). Specifically, the cells were collected and then lysed with kit lysis buffer on ice for 15 min; resulting homogenate was centrifuged at 18,000 × g (15 min, 4°C). The protein content in each cleared supernatant sample was determined using a Bradford protein assay kit (Beyotime). Subsequently, the samples were diluted with PBS such that equivalent amounts of total protein could be present in a given-size aliquot. For the assay, 50 µL supernatant (containing 100 µg total protein) was transferred into a dedicated well in a 96-well plate; to each well, 40 µL kit reaction buffer and 10 µL 2 mM caspase-1 substrate were added and the plate was incubated at 37°C for 2 h. The amount of yellow formazan pNA in each well was then measured at 405 nm in the microplate reader. Caspase-1 activity was calculated as per manufacturer instructions, and all outcomes were presented as fold-change in activity compared to in the control cells.

### Enzyme-linked immunosorbent assay (ELISA)

The culture supernatant that was collected was stored at −80°C until analyed. ELISA was performed using specific kits for IL-1β and IL-18 (MLBIO, Shanghai, China) per manufacturer instructions. Optical density in each kit well was measured at 450 nm using the microplate reader. Each sample was analyzed in triplicate. The limit of sensitivity of each kit was 7.81–500 pg IL-1 β/mL and 15.63–1,000 pg IL-18/mL.

### Statistics

All data processing and statistical analyses were performed using IBM SPSS Statistics (v.22.0, IBM Corp., Armonk, NY). Data are expressed as means ± SE. Before performing statistical tests, all data were tested for normality using the Shapiro-Wilk test. For data that followed a normal distribution, an analysis of variance (ANOVA) was performed, and an independent sample t-test was used to compare the groups. If normality assumptions were not met, non-parametric tests such as the Mann-Whitney U test (for two groups) or Kruskal–Wallis test (for more than two groups) were applied. Post-hoc analysis was conducted using either a Student’s t-test (for comparing two groups) or a least significant difference (LSD) test (for comparing more than two groups). Differences were considered statistically significant at *p < 0.05 or **p < 0.01.

## Results

### Interaction between cordycepin and NLRP3, Caspase-1, GSDMD proteins

**FIGURE 1:**
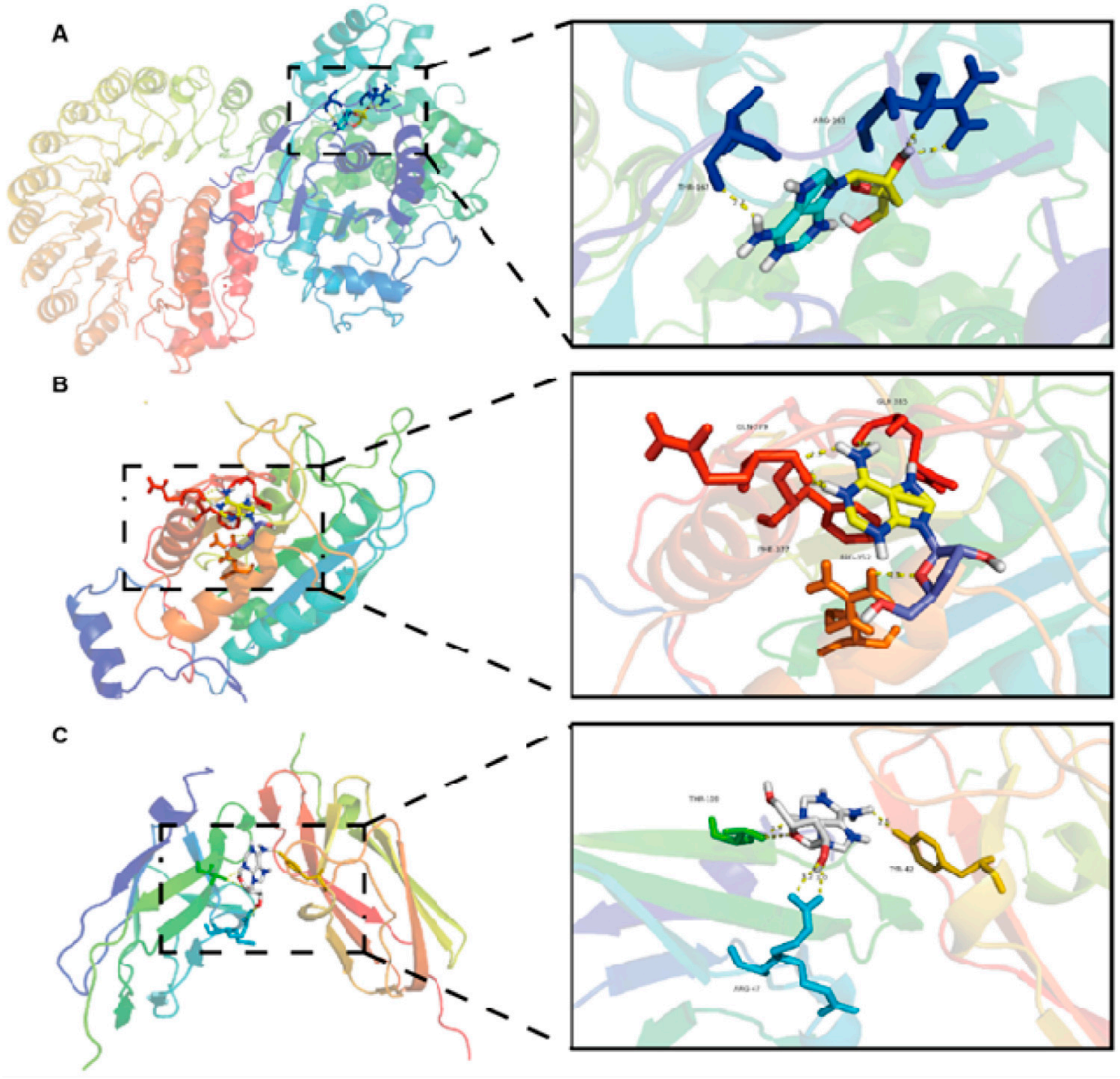
Molecular docking model of cordycepin and the pyrodeath pathway signaling protein.

It is generally believed that the lower the binding energy between small molecule ligands and protein receptors, the more stable the binding conformation. When the binding energy between the two is ≤ −5.0 kcal/mol, it indicates that they have good binding activity. The results of the interaction between cordycepin and NLRP3, Caspase-1, GSDMD proteins are shown in Table 2. The binding energies of cordycepin with the NLRP3, Caspase-1, and GSDMD proteins in the pyroptosis signaling pathway were all < −5 kcal/mol, indicating cordycepin had a strong binding ability with the three key proteins in the pyroptosis signaling pathway. When the docking results were imported into PyMOL 1.8.x software for visualization (Figure 1) it was observed that the binding of cordycepin to the target proteins occured in the form of hydrogen bonds. Figure 1 illustrates that cordycepin formed hydrogen bonds with the amino acid residues THR-169 and ARG-165 on the NLRP3 protein, with residues GLN-379, GLN-385, PHE-377, and ARG-352 on caspase-1, and with residues VAL-58, ALA-56, ILE-48, and ILE-46 on the GSDMD protein.

**TABLE 2:** Docking results of cordycepin with three main target proteins of pyrodeath signaling pathway.

Target proteins	PDB ID	Binding energy (Kcal/mol)	Amino acid residues interacting with cordycepin
NLRP3	6NPY	−6.90	THR-169、ARG-165
Caspase-1	5NHI	−5.80	GLN-379、GLN-385、PHE-377、ARG-352
GSDMD	IRWM	−5.21	VAL-58、ALA-56、ILE-48、ILE-46

### Cordycepin inhibits the production of ROS in macrophages

**FIGURE 2:**
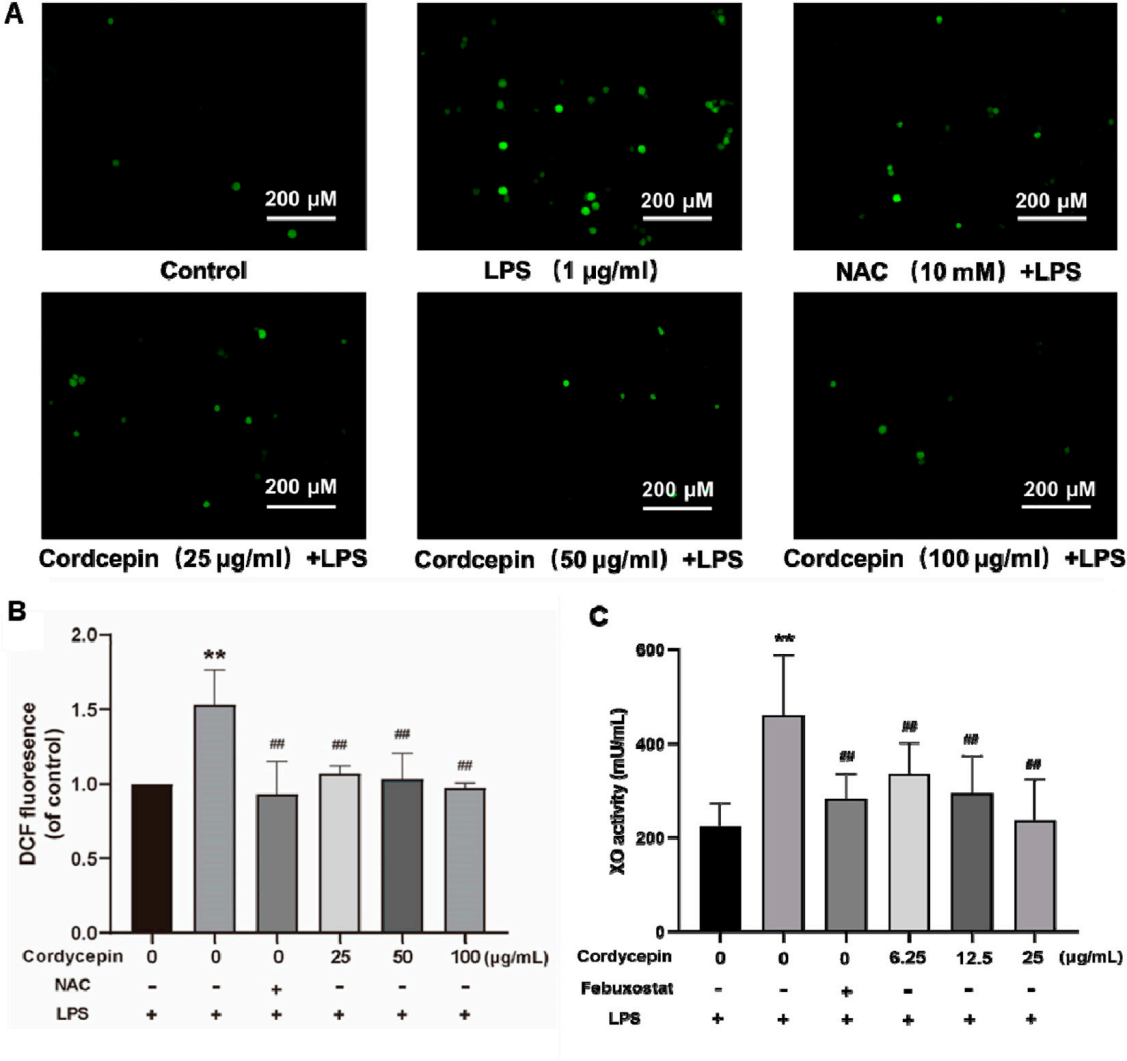
Cordycepin inhibits LPS-induced ROS and XO generation in mouse RAW264.7 macrophages. **(A)** Fluorescence microscopy. **(B)** Fluorescence microplate readings. **(C)** Micro-plate detection of ROS (mainly from XO). Compared to sham group, **p < 0.01; compared to LPS-only group, ^##^p < 0.01. n = 3 per group.

Reactive oxygen species (ROS) can act as signaling molecules, including those that activate the NLRP3 inflammasome to participate in cell pyroptosis. In macrophages, xanthine oxidase (XO) is a main source for generation of ROS. As expected, XO activity in mouse RAW264.7 cells is stimulated by lipopolysaccharide (LPS), leading to excess ROS production. Therefore, to assess if cordycepin could impact on RAW264.7 cell formation of ROS, levels of ROS and XO activity were evaluated. The results show that compared to in sham cells, the expression level of ROS in LPS-stimulated macrophages was significantly increased (Figure 2).

*N-acetylcysteine* (NAC) was used as a positive control in this study due to its ability to inhibit ROS production and suppress NLRP3 inflammasome activation, serving to validate the anti-pyroptosis effects of cordycepin. In comparison to the LPS group, intervention with the NAC or different concentrations of cordycepin resulted in a significant decrease in the expression levels of ROS. Here, even use of the lowest concentration of cordycepin (i.e., 25 μg/mL) led to significant decreases in ROS that could be induced by LPS. Therefore, in subsequent experiments, the dosage of cordycepin was adjusted to 6.25, 12.5, and 25 μg/mL.

The XO activity results showed that compared to in the sham group, XO activity in LPS- stimulated RAW264.7 cells was significantly increased (Figure 2). Studies have reported that the XO inhibitor febuxostat can significantly reduce XO-induced excessive ROS production (Kim et al., 2020; Higa et al., 2023). To verify that here, cells were treated with this drug and XO activity assessed. In comparison to the LPS group, treatment of macrophages with febuxostat resulted in a significant decrease in XO activity. These results were similar overall to those with cordycepin wherein XO activity in the cells was significantly inhibited in what appeared to be a dose-related manner.

### LPS alone up-regulated RAW264.7 expression of XO, NLRP3, Caspase-1, and GSDMD

**FIGURE 3:**
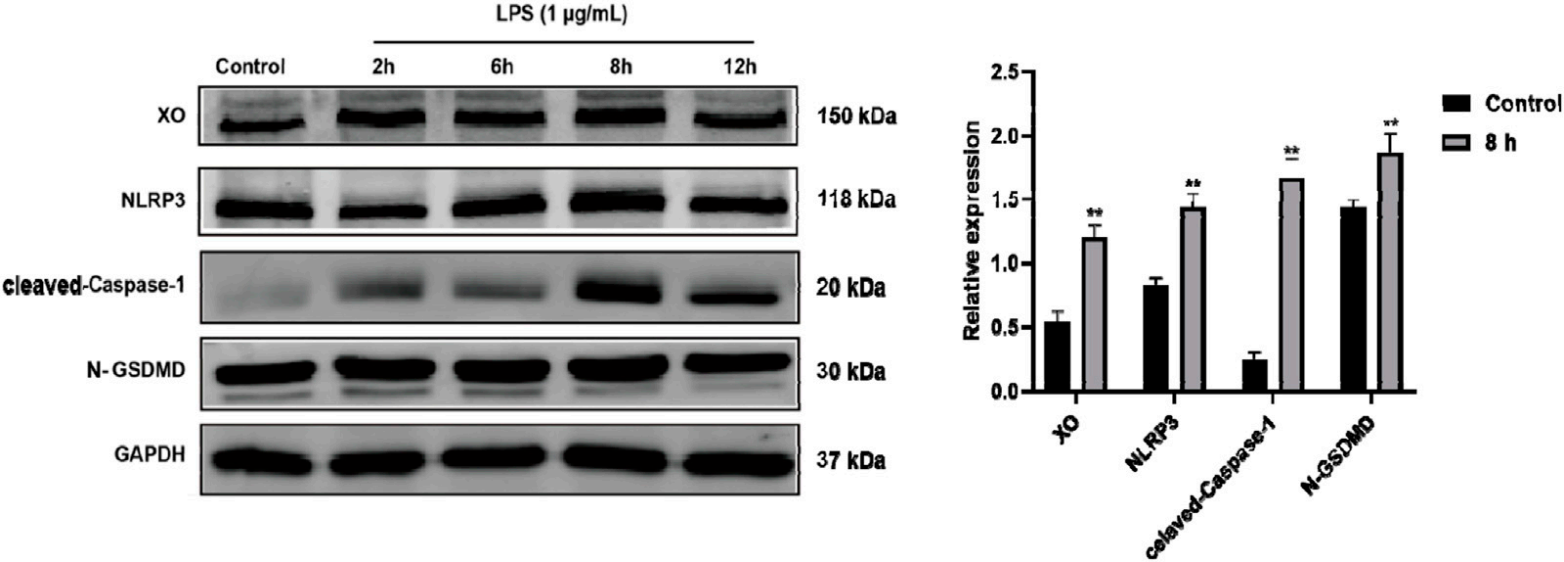
Time-related LPS-induced expression of XO, NLRP3, Caspase-1, and GSDMD proteins in RAW264.7 macrophages. Representative blots are shown. Compared to Control group, **p < 0.01. n = 3 per group.

The docking experiments showed that cordycepin had a strong binding ability with pyroptosis-related NLRP3, Caspase-1, and GSDMD proteins. To determine the timeline of effect on the expression of these proteins, macrophages were LPS-stimulated for 2, 6, 8, and 12 h and changes in expression levels of NLRP3, cleaved-Caspase-1, and cleaved N-terminal GSDMD proteins were monitored using Western blot. As shown in Figure 3, LPS stimulation resulted in increased expression of each protein in the macrophages. Expression levels of XO, NLRP3, cleaved-Caspase-1, and cleaved N-terminal GSDMD proteins reached their highest level at 8 h of stimulation.

### Cordycepin effects on macrophages of *Xo, Nlrp3, Caspase-1,* and *Gsdmd* mRNA

**FIGURE 4:**
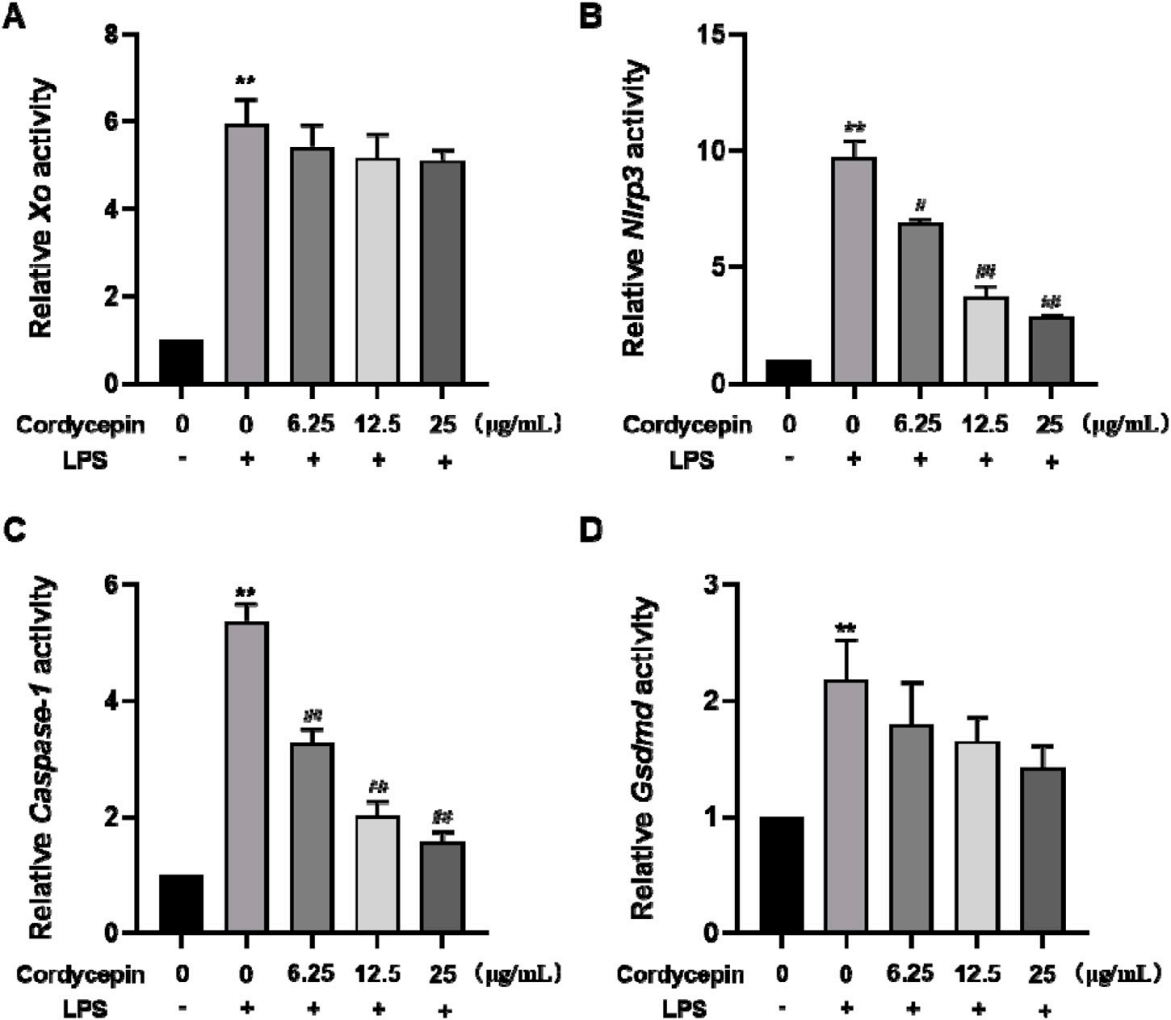
Effects of cordycepin on relative activity of **(A)** XO, **(B)** NLRP3, **(C)** Caspase-1, and **(D)** GSDMD in LPS-treated RAW264.7 macrophages. Compared to sham group, **p < 0.01; compared to LPS-only group, ^#^p < 0.05; ^##^p < 0.01. n = 3 per group.

To ascertain if changes in the protein expression of the four proteins occurred at the translational level, qRT-PCR experiments were performed to assess expression levels of *Xo*, *Nlrp3*, *Caspase-1*, and *Gsdmd* mRNA in the RAW264.7 macrophages. The experimental results, indicate that compared to in the sham group, mRNA levels of *Xo*, *Nlrp3*, *Caspase-1*, and *Gsdmd* in LPS-stimulated mouse RAW264.7 macrophages significantly increased (Figure 4). Interestingly, compared to in the LPS-only group, cells with different doses of cordycepin showed no significant changes in *Xo* and *Gsdmd* mRNA expression levels, but did show significantly inhibited expression of *Nlrp3* and *Caspase-1* mRNA.

### Cordycepin affected macrophage XO, NLRP3, cleaved-Caspase-1, and cleaved N-terminal GSDMD protein expression

**FIGURE 5:**
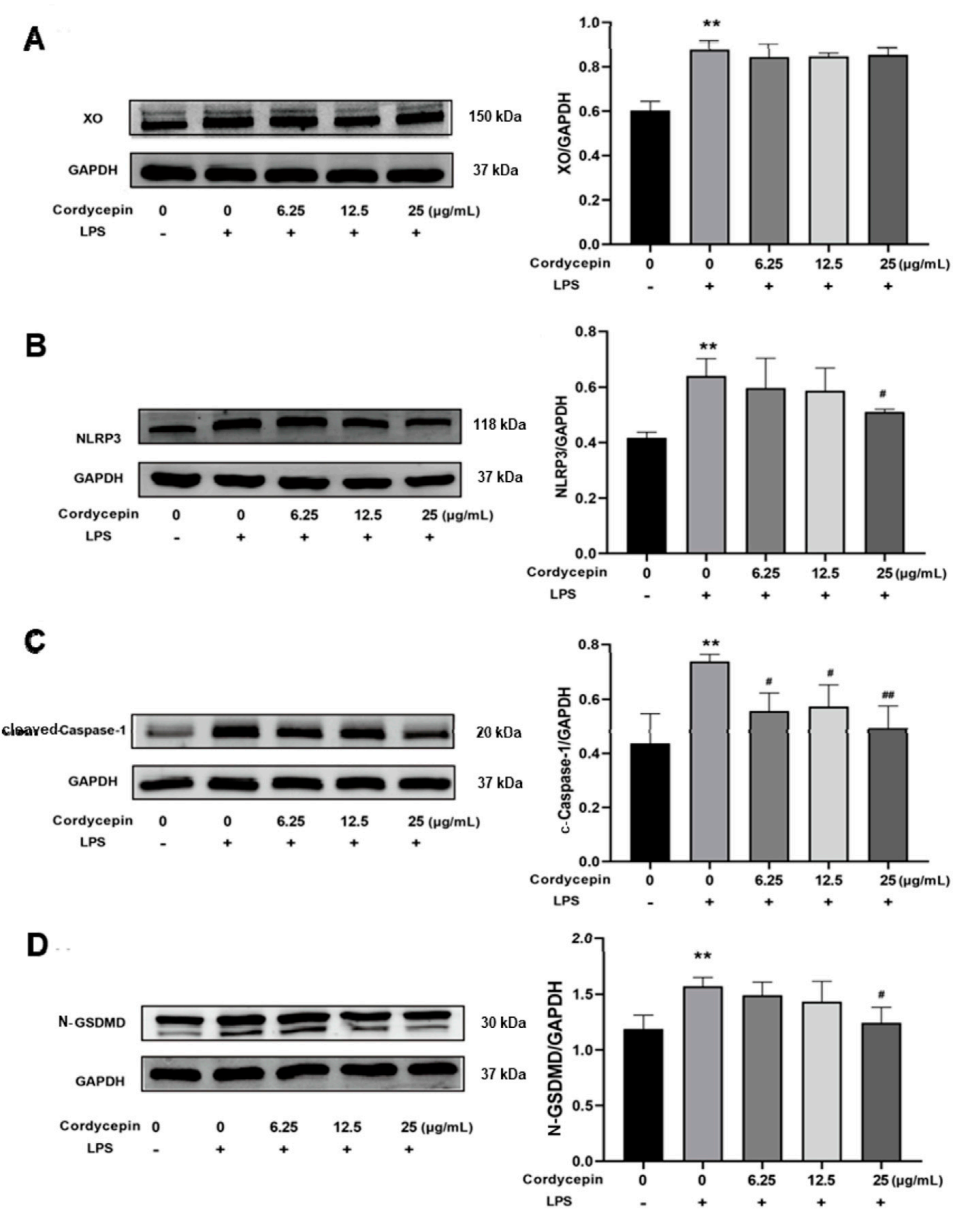
Effects of cordycepin on protein (left) and mRNA (right) expression of **(A)** XO, **(B)** NLRP3, **(C)** Caspase-1, and **(D)** GSDMD in LPS-treated RAW264.7 macrophages. Compared to sham group, **p < 0.01; compared to LPS-only group, ^#^p < 0.05; ^##^p < 0.01. n = 3 per group.

To determine if similar effects as seen with mRNA occurred with respect to expression of XO, NLRP3, cleaved-Caspase-1, and cleaved N-terminal GSDMD proteins, Western blot assays were performed. As expected, in the LPS-stimulated macrophages, expression of each was significantly increased (Figure 5). Compared to in the LPS-only group, treatment with different doses of cordycepin had no significant effect on expression level of XO protein. Treatment with 6.25 or 12.5 μg cordycepin/mL downregulated the expression of NLRP3 protein in a non-significant manner, but the 25 μg/mL dose caused a significant reduction. Treatment with any of the three concentrations of cordycepin all significantly downregulated cleaved-Caspase-1 protein expression levels. With regard to cleaved N-terminal GSDMD protein, treatments with either 6.25 or 12.5 μg cordycepin/mL treatment groups caused a weak downregulation trend in, but the effect was not significant. As with NLRP3, use of the 25 μg cordycepin/mL caused a significant reduction in cleaved N-terminal GSDMD expression levels vs. that in zLPS-only cells.

### Cordycepin inhibited macrophage pyroptosis

**FIGURE 6:**
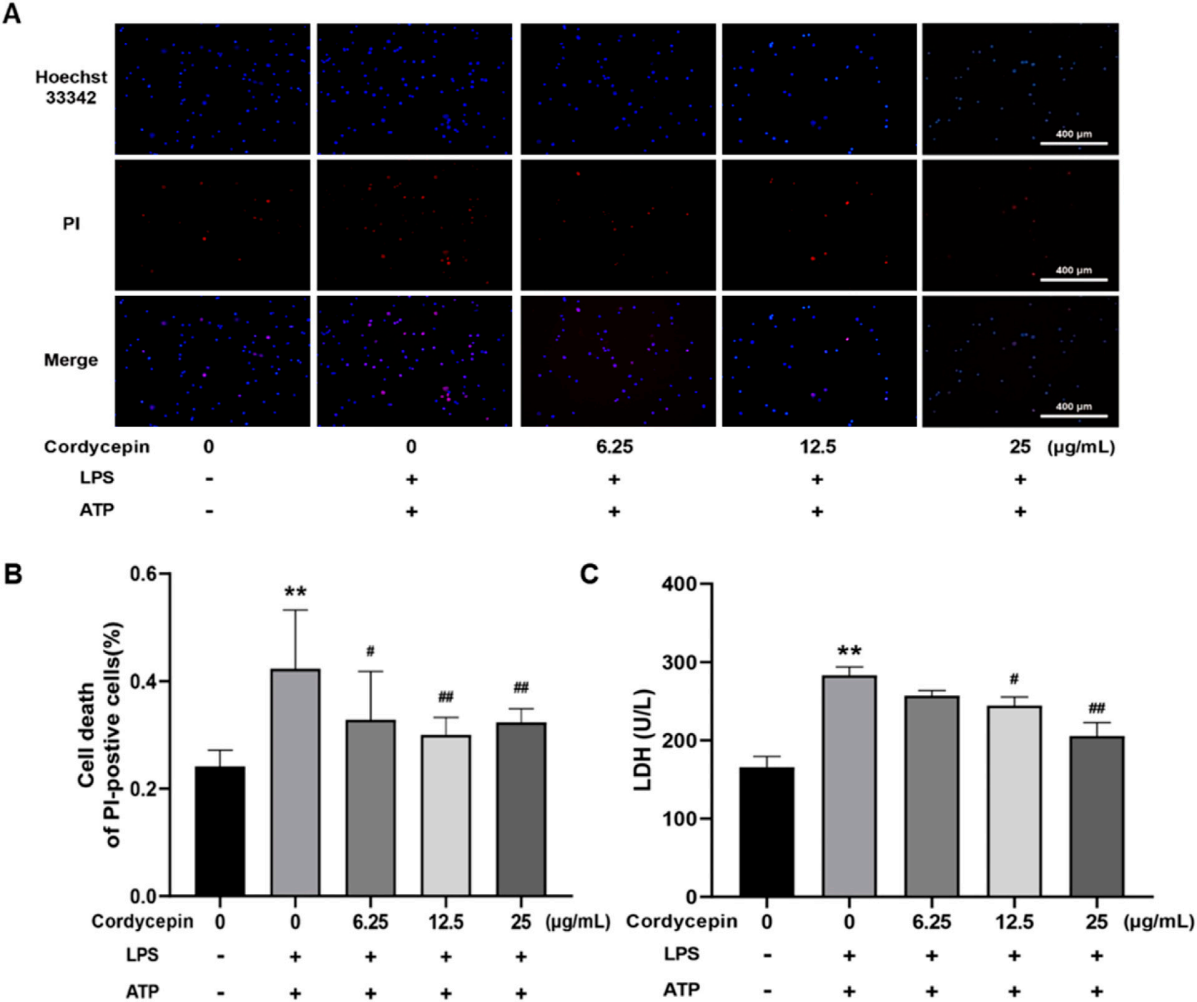
Cordycepin effects on cell death rate induced by LPS and ATP. **(A)** Staining under fluorescence microscope (100x). **(B)** Numbers of PI^+^ cells. **(C)** Effect of cordycepin on LDH release of RAW264.7 cell stimulated by LPS + ATP. Compared to sham group, **p < 0.01; compared to LPS-only group, ^#^p < 0.05; ^##^p < 0.01. n = 3 per group.

Results of the double-staining experiments with PI and Hoechst 33,342 indicated that, compared to the sham group, macrophages stimulated with LPS + ATP had a significant increase in red fluorescence intensity and in percentages of PI^+^ cells (Figures 6A, B). In contrast, Cordycepin inhibited macrophage cell death, as indicated by a significant decrease in PI + percentages. The LDH release assay results (Figure 6C) also revealed that compared to the sham group, LDH release by the macrophages stimulated with LPS (combined with ATP) significantly increased. Again, treatment with different concentrations of cordycepin significantly reduced LDH release, with the effect being seen to occur in a cordycepin concentration-dependent manner. This outcome indicated that cordycepin had an inhibitory effect on cell membrane permeabilization.

### Cordycepin inhibited macrophage caspase-1 activity

**FIGURE 7:**
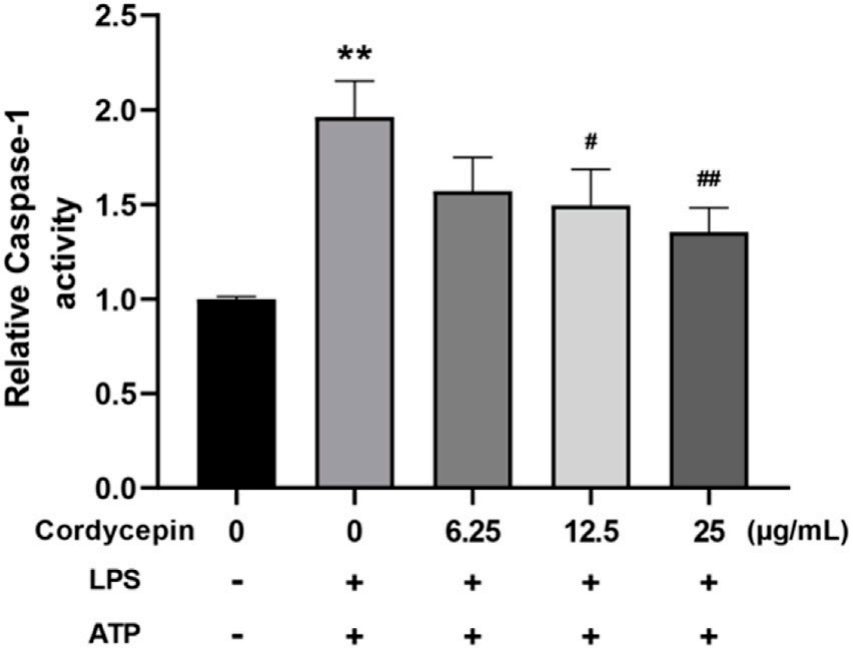
Cordycepin effects on caspase-1 activity in LPS/ATP-treated RAW264.7 macrophages. Compared to sham group, **p < 0.01; compared to LPS-only group, ^#^p < 0.05; ^##^p < 0.01. n = 3 per group.

Caspase-1 is a key protease in the process of pyroptosis and a hallmark of the classical pyroptosis pathway is activation of caspase-1. Activated caspase-1 can cleave the GSDMD protein and pro-IL-1β and pro-IL-18, leading to formation of pores on the cell membrane and the maturation/release of inflammatory IL-1β and IL-18. To assess potential effects of cordycepin on this aspect of pyroptosis in macrophages, caspase-1 activity assays were undertaken. The results indicate that compared to in sham cells, treatment of the RAW264.7 cells with LPS + ATP led to increased intracellular caspase-1 enzyme activity (Figure 7). Compared to in this LPS + ATP group, cordycepin treatment group significantly inhibited intracellular caspase-1 activity, and the degree of inhibition was positively correlated with the concentration of cordycepin. This demonstrated that while LPS + ATP stimulation could activate originally inactive caspase-1 enzyme in macrophages, treatment with cordycepin inhibited/mitigated this activation.

### Cordycepin inhibits macrophage expression and release of IL-1β and IL-18

**FIGURE 8:**
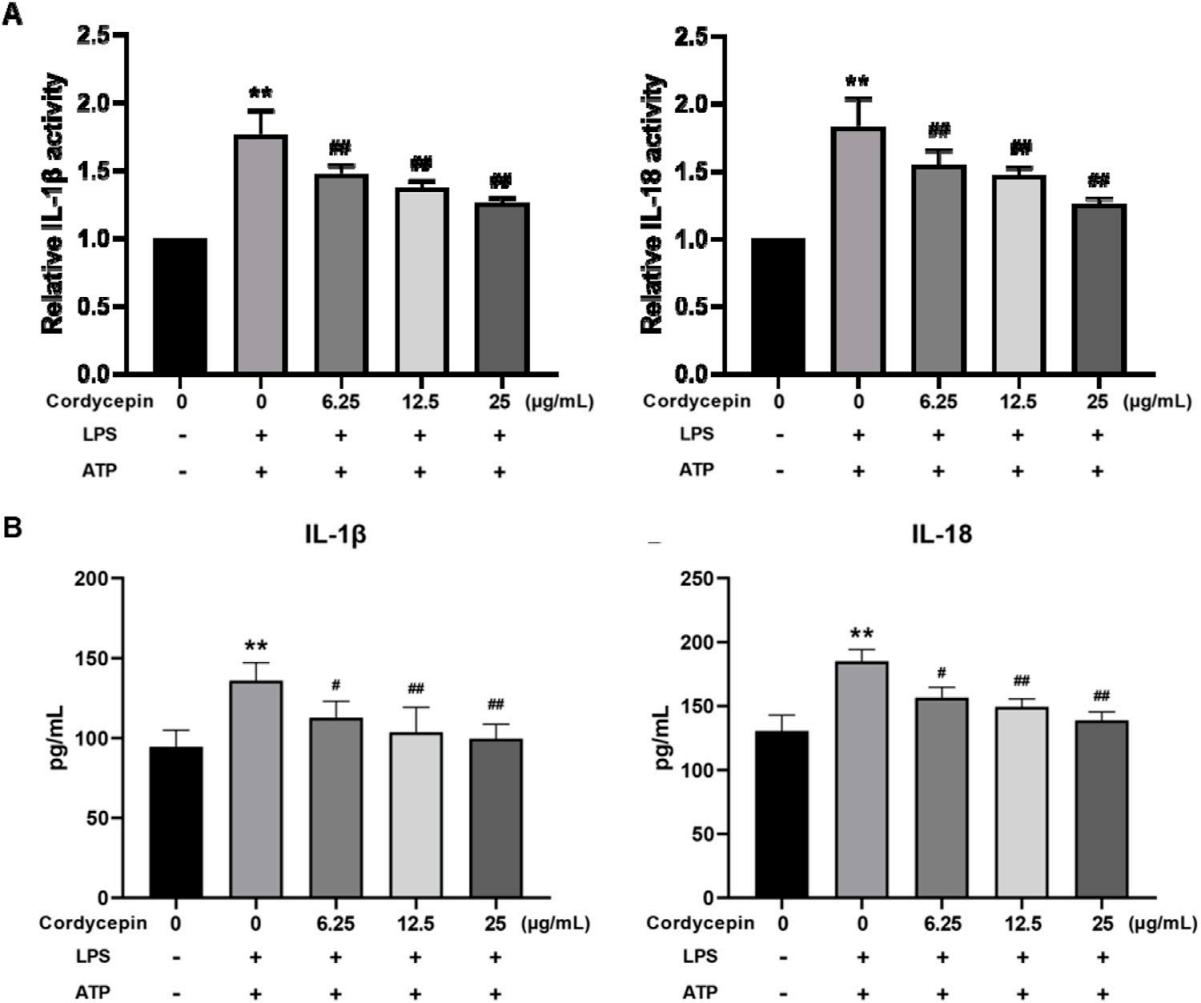
Effect of cordycepin on IL-1β and IL-18 **(A)** expression, and **(B)** secretion by LPS + ATP-treated RAW264.7 macrophages. Compared to sham group, **p < 0.01; compared to LPS-only group, ^#^p < 0.05; ^##^p < 0.01. n = 3 per group.

Based on the above outcomes, it was expected that there would be changes in the LPS inducibility of RAW264.7 macrophages regarding IL-1β and IL-18. To confirm this, we evaluated both the mRNA expression levels and the release of these cytokines. Using qPCR, we observed that compared to the sham group, LPS + ATP stimulation significantly increased IL-1β and IL-18 mRNA expression in RAW264.7 cells. In addition, ELISA results showed that the release of IL-1β and IL-18 into the culture supernatant was also significantly increased (Figure 8). Notably, treatment with different concentrations of cordycepin significantly inhibited both the mRNA expression and the release of IL-1β and IL-18 in a dose-dependent manner. These consistent results further support that cordycepin can suppress the downstream inflammatory responses, including the production and release of IL-1β and IL-18, during macrophage pyroptosis.

## Discussion

Macrophages are an important component of innate immunity and have always been at the core of immunological research into various processes such as inflammation, immune responses, and tissue repair within the body (Kelly and O’Neill, 2015), as well as reactions to invading pathogens. Macrophages are a key source of inflammatory mediators in a host, serving as key effector cells in the innate immune response and inflammation when the body encounters infections (Bäck et al., 2019). In general, inflammation is a self-protective response that both is critical in killing pathogens as well as in helping a host maintain immune homeostasis.

When there is an imbalance in macrophage/immune function, inflammation can persist to the point of causing pathologies. Long-term chronic inflammation can lead to tissue damage and potentially organ failure. Currently, the most commonly used anti-inflammatory therapies in clinical practice include traditional corticosteroids and non-steroidal anti-inflammatory drugs (NSAIDs). However, these medications not only have serious side effects but also do not have long-term good efficacies in treating chronic inflammatory diseases (Eirefelt et al., 2022). Studies have shown that certain traditional Chinese medicines can effectively treating chronic inflammation (Chan and Ng, 2020; Li, 2020; Wang et al., 2021). However, the cellular and molecular mechanisms by which many these medicines exert their effects are still largely unknown. This is particularly the case for cordycepin.

Studies have shown that over-activated macrophages can produce more pro-inflamma- tory cytokines, thereby promoting the occurrence of inflammation or exacerbating autoimmune diseases (Xiang et al., 2023). To better understand how these cells may become inadvertently malevolent, many *in vitro* model systems have been utilized. Among these, the lipopolysaccharide (LPS)-induced RAW264.7 macrophage inflammation model has been widely used in studies of inflammatory responses (Fang et al., 2020). From those types of studies, it has been made clear that during immune activation, excessive macrophage production of reactive oxygen species (ROS) is crucial for triggering several cell signaling cascades (Sarmiento-Salinas et al., 2021). It is also now known that these increases in ROS formation can promote the activation of the NLRP3 inflammasome which in turn can lead to cell pyroptosis (Wu et al., 2020).

**FIGURE 9:**
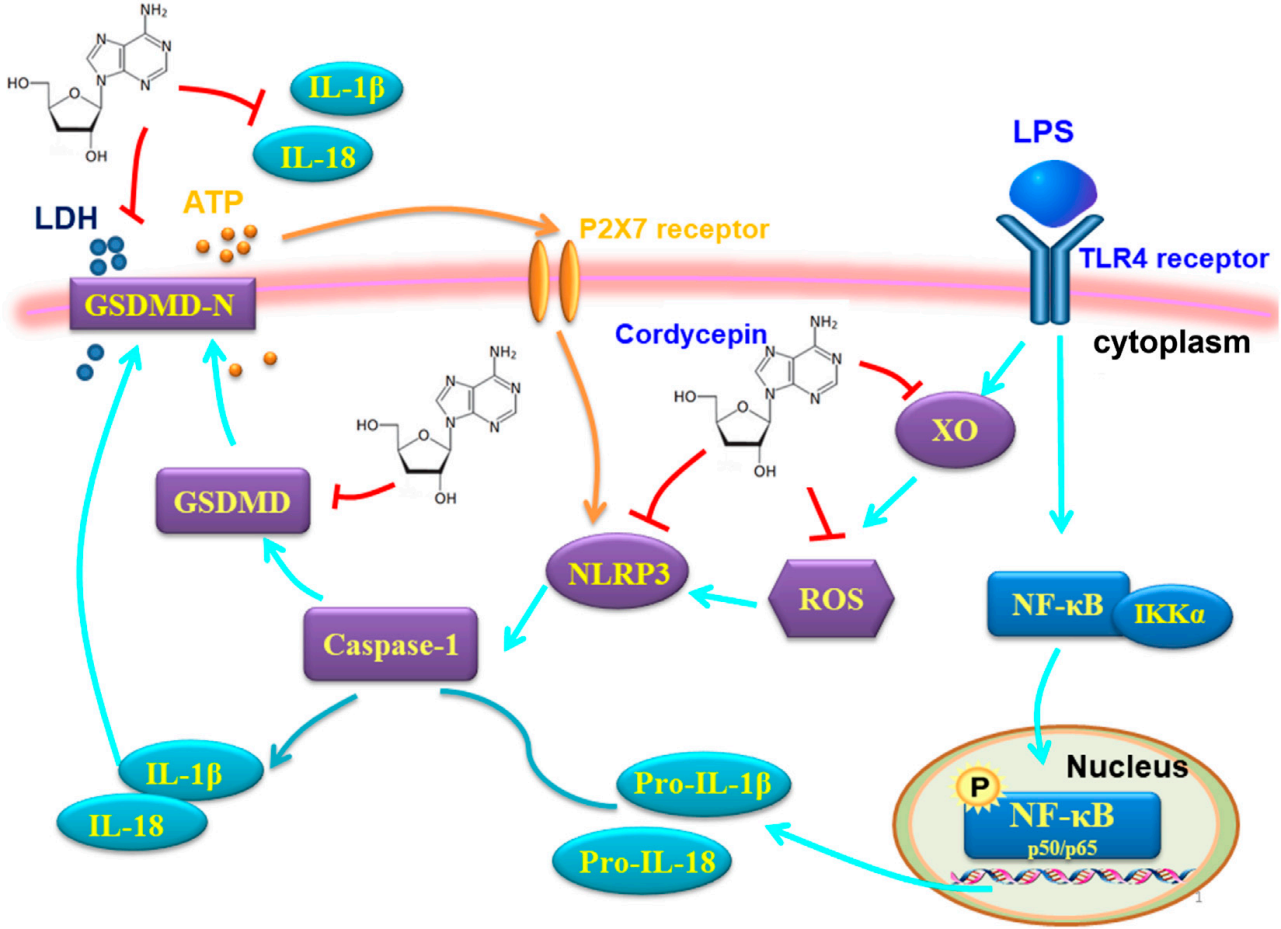
Effect and mechanism of cordycepin on macrophage pyroptosis function.

To begin to define potential mechanisms by which cordycepin might impact macrophage functional characteristics *in vitro*, the study here used the RAW264.7 macrophage model noted above. Initially, molecular docking technology was used to investigate the ability of cordycepin to bind three key protein targets in the macrophage pyroptosis signaling pathway. The docking results showed that cordycepin had an affinity of < −5 kcal/mol with all three key pyroptosis targets, indicating a strong binding ability. This suggested then that these proteins may be key targets for cordycepin when it exerts any anti-pyroptosis effect. Cordycepin’s water solubility enables efficient delivery *in vitro* systems, facilitating its biological activity and experimental application. A graphical summary of the molecular mechanisms by which cordycepin impacts the macrophage pyroptosis signaling pathway is shown in Figure 9. As illustrated, cordycepin inhibits key steps in the pathway, including ROS production, NLRP3 activation, and downstream pyroptosis-related processes, thereby reducing the release of IL-1β and IL-18.

Subsequently, it was also shown here that cordycepin significantly reduced LPS- stimulated production of ROS by the RAW264.7 macrophages. This was the case even with the lowest concentration of cordycepin tested (i.e., 25 μg/mL). It was also seen that XO - one of the main enzyme sources of ROS in a macrophage in response to stimuli (such as LPS and cytokines) (Liu et al., 2021), was affected by cordycepin. Using qRT-PCR and Western blot assays to examine gene and protein levels (respectively) of key proteins, it was shown that cordycepin significantly down-regulated expression of the genes and proteins for pyroptosis-related NLRP3, Caspase-1, and GSDMD. The most significant inhibitory effect on NLRP3, Caspase-1, and GSDMD genes and proteins was seen at a concentration of 25 μg cordycepin/mL. These outcomes are in keeping with other literature reports that have shown how various natural compounds can inhibit activation of NLRP3 inflammasomes and down-regulate pyroptosis-related proteins to suppress inflammation (Tang et al., 2019; Zu et al., 2019; Li et al., 2021). From these results, while one might then conclude that targeting regulation of NLRP3 inflammasome activation and pyroptosis as a means to treat inflammation-related diseases may be a novel therapy, it is still not known whether cordycepin can inhibit the ultimate downstream effects of macrophage pyroptosis caused by NLRP3 activation.

Research has found that LPS binds its membrane receptor TLR4, activates the NF-κB signaling pathway, and induces expression of NLRP3, pro-IL-1β, and pro-IL-18 (Qu et al., 2020; Huang et al., 2021). Extracellular ATP acts as a second enhancing signal for sustained activation of NLRP3 inflammasomes, which in turn can trigger intracellular K^+^ efflux by binding to P2X7R, activating the NLRP3 inflammasome, further inducing downstream cell pyroptosis (Franceschini et al., 2015). Hoechst 33,342 and PI staining experiments here revealed that the macrophages pre-treated with different levels of cordycepin had significant decreases in red fluorescence intensity and in percentages of PI^+^ cells. These outcomes indicated that cordycepin could inhibit pyroptosis of these macrophages. In the LPS + ATP stimulation group, the LDH release rate and PI^+^ percentages among the macrophages significantly decreased, and cordycepin significantly inhibited LDH release rate and PI^+^ percentages, suggesting to us that cordycepin imparted some capacity for cell membrane integrity to recover.

Both IL-1β and IL-18 are the most common inflammatory factors when cell pyroptosis occurs (Sahoo et al., 2011). They can be cleaved by activated Caspase-1 to form active IL-1β and IL-18, which are then released into the extracellular space as the cell membrane ruptures (Kordes et al., 2011). The massive release of inflammatory factors leads to recruitment of surrounding inflammatory cells, further amplifying the local inflammatory response. In the study here, measures of the expression and release of IL-1β and IL-18 into the cell culture supernatant showed that cordycepin significantly inhibited expression of both cytokines by the LPS/ATP-induced RAW264.7 cells. From this, it might be concluded that cordycepin can exert an anti-inflammatory effect in part by inhibiting macrophage pyroptosis. The results here also showed that cordycepin could improve the condition of pyroptotic cells in terms of mortality and cell membrane integrity. Therefore, it might be assumed that among its many biological effects, cordycepin can inhibit macrophage pyroptosis.

Though there are several significant findings from the studies here, it is important to still noted that there are certain limitations to this study. Firstly, the experiments were conducted using only a single macrophage cell line (RAW264.7), which may not be the most optimal model for studying NLRP3 inflammasome activation. Additional cell lines, such as bone marrow-derived macrophages (BMDMs) or THP-1-derived macrophages, could provide further confirmation of our findings. Secondly, the research was performed only at the cellular level *in vitro*; the lack of *in vivo* models limits the translational relevance of our results. Future studies using LPS-induced sepsis models or inflammatory bowel disease models are necessary to validate the anti-inflammatory effects of cordycepin in physiological conditions. Thirdly, while existing literature has shown that the ROS inhibitor NAC can inhibit NLRP3 activation, direct comparisons between cordycepin and NAC in inhibiting macrophage pyroptosis were not conducted. Fourthly, it is necessary to investigate and confirm whether cordycepin also has inhibitory effects on pyroptosis in other types of macrophages. Additionally, although this study focused on the downstream molecular mechanisms of ATP-P2X7R signaling, we did not assess the density or expression level of P2X7 receptors on the cell surface. Future studies could investigate the role of P2X7R expression in modulating NLRP3 inflammasome activation and pyroptosis, which would provide further insights into the mechanism of cordycepin’s action.

The relationship between cordycepin and other signaling pathways related to macrophage pyroptosis has not been deeply explored. With the increasing incidence of inflammatory diseases, development of anti-inflammatory/immune-modulating drugs has become increasingly important in new drug research and development. Currently, molecular mechanisms of cordycepin in inhibiting inflammation and immunoregulation are being continuously elucidated. It is hoped that in time, based on findings here and from other investigators, cordycepin may become more accepted as an anti-inflammatory drug.

## Data Availability

The original contributions presented in the study are included in the article/Supplementary Material, further inquiries can be directed to the corresponding authors.
