# Supplementary figures and images for: Cordycepin attenuates NLRP3/Caspase-1/GSDMD-mediated LPS-induced macrophage pyroptosis
Source: Front Pharmacol. 2025 Feb 14;16:1526616. doi: 10.3389/fphar.2025.1526616 (PMC11868043; doi:10.3389/fphar.2025.1526616)

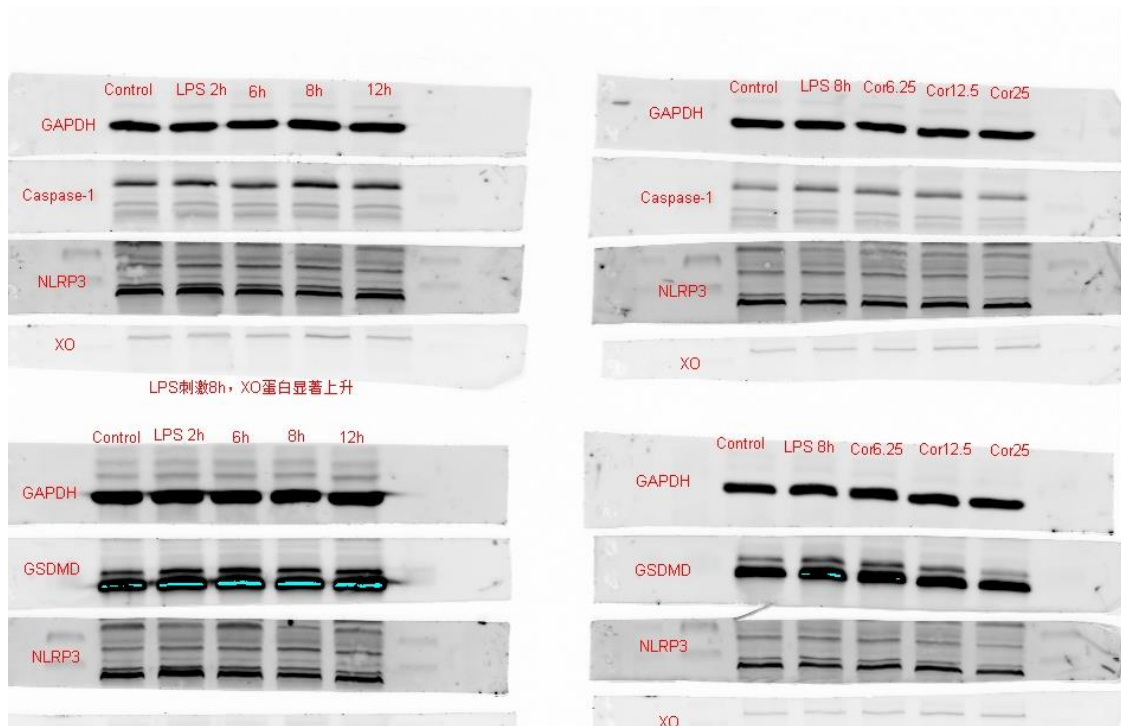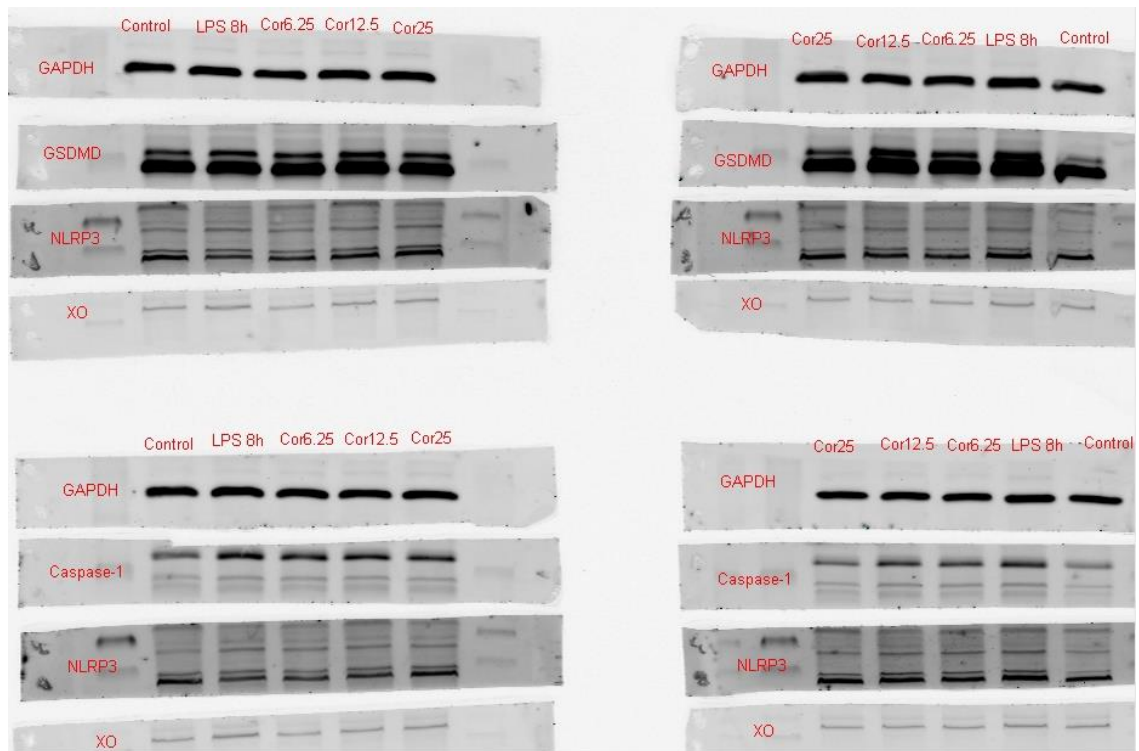

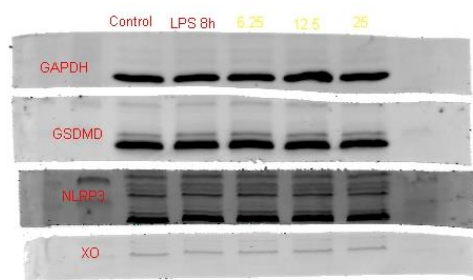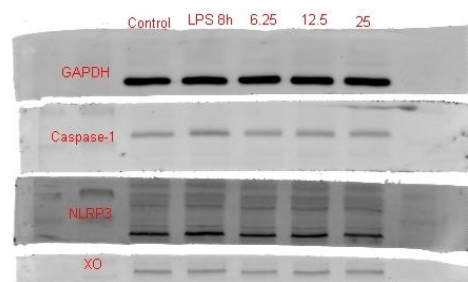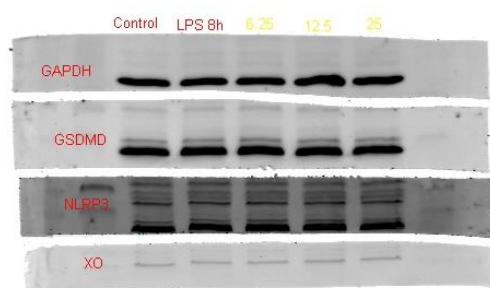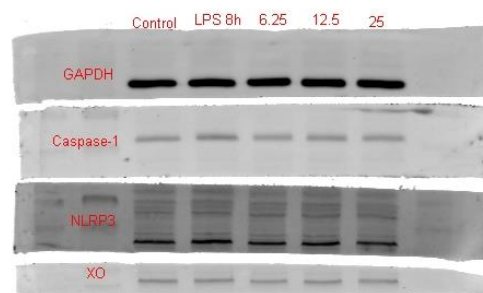

Supplement: Supplementary file 1 [file DataSheet1.pdf]
